# Treatment outcomes and associated factors for hospitalization of children treated for acute malnutrition under the OptiMA simplified protocol: a prospective observational cohort in rural Niger

**DOI:** 10.3389/fpubh.2023.1199036

**Published:** 2023-07-05

**Authors:** Kevin Phelan, Benjamin Seri, Maguy Daures, Cyrille Yao, Rodrigue Alitanou, Ahmad Ag Mohamed Aly, Oumarou Maidadji, Atté Sanoussi, Aboubacar Mahamadou, Cécile Cazes, Raoul Moh, Renaud Becquet, Susan Shepherd

**Affiliations:** ^1^The Alliance for International Medical Action (ALIMA), Dakar, Senegal; ^2^PRISME-CI ANRS|MIE Research Programme, University Hospital of Treichville, Abidjan, Côte d'Ivoire; ^3^National Institute for Health and Medical Research (INSERM) UMR 1219, Research Institute for Sustainable Development (IRD) EMR 271, Bordeaux Population Health Research Centre, University of Bordeaux, Bordeaux, France; ^4^The Alliance for International Medical Action (ALIMA), Niamey, Niger; ^5^Bien Etre de la Femme et de l'Enfant (BEFEN), Niamey, Niger; ^6^Ministry of Health, Nutrition Division, Niamey, Niger; ^7^High-Commission of the Nigériens Nourrissent les Nigériens (3N) Initiative, Niamey, Niger; ^8^Dermatology and Infectiology Pedagogical Unit, Training and Research Units in Medical Sciences, Abidjan, Côte d'Ivoire

**Keywords:** mid-upper arm circumference (MUAC), acute malnutrition, hospitalization, West Africa, children, small-quantity lipid-based nutrient supplements (SQ-LNS)

## Abstract

**Introduction:**

Globally, access to treatment for severe and moderate acute malnutrition is very low, in part because different protocols and products are used in separate programs. New approaches, defining acute malnutrition (AM) as mid-upper arm circumference (MUAC) < 125 mm or oedema, are being investigated to compare effectiveness to current programs. Optimizing Malnutrition treatment (OptiMA) is one such strategy that treats AM with one product – ready-to-use therapeutic food, or RUTF – at reduced dosage as the child improves.

**Methods:**

This study aimed to determine whether OptiMA achieved effectiveness benchmarks established in the Nigerien National Nutrition protocol. A prospective cohort study of children in the rural Mirriah district evaluated outcomes among children 6-59 months with uncomplicated AM treated under OptiMA. In a parallel, unconnected program in one of the two trial sites, all non-malnourished children 6-23 months of age were provided small quantity lipid-based nutritional supplements (SQ-LNS). A multivariate logistic regression identified factors associated with hospitalization.

**Results:**

From July-December 2019, 1,105 children were included for analysis. Prior to treatment, 39.3% of children received SQ-LNS. Recovery, non-response, and mortality rates were 82.3%, 12.6%, and 0.7%, respectively, and the hospitalization rate was 15.1%. Children who received SQ-LNS before an episode of AM were 43% less likely to be hospitalized (ORa=0.57; 0.39-0.85, *p* = 0.004).

**Discussion:**

OptiMA had acceptable recovery compared to the Nigerien reference but non-response was high. Children who received SQ-LNS before treatment under OptiMA were less likely to be hospitalized, showing potential health benefits of combining simplified treatment protocols with food-based prevention in an area with a high burden of malnutrition such as rural Niger.

## 1. Introduction

An estimated 45.4 million children under 5 are affected by wasting worldwide, of which 31.8 million are moderately wasted and 13.6 million are severely wasted (1). Relying on prevalence rather than incidence, however, likely underestimates the true number of children affected (2). Disruptions from the Covid-19 pandemic have increased the global burden of malnutrition, particularly in the Sahel region of Africa (3).

Even though treatment protocols for moderate acute malnutrition (MAM) and severe acute malnutrition (SAM) have been scaled-up since 2007, less than one fourth of children have access to treatment (4). This is in part due to the cumbersome nature of the current approach in which MAM and SAM are treated separately, using different protocols and products in programs overseen by different UN agencies. To address these shortcomings, current research is focusing on ways to decentralize treatment, integrate MAM and SAM management, and reduce the dosage of ready to use therapeutic foods (RUTF) during treatment (5–8). Because the drivers and epidemiology of malnutrition are context-specific, it will be important to test such protocols in multiple countries.

Most children with SAM and MAM can be treated on an outpatient basis (9). However, an estimated 15% of children with SAM present with complications that require hospital care (In an email from K. de Polnay, M.D., Médecins Sans Frontières^1^ on December 17, 2020). These children are at an elevated risk of mortality. According to the World Health Organization (WHO), less than 10% of children with complicated SAM will die if inpatient management guidelines are followed (10). But a recent meta-analysis found the mean inpatient mortality is greater than 15%, with some programs reporting rates higher than 40% (11). There are no estimates for the percentage of children with MAM requiring hospitalization, but a recent study has shown that there is a substantial burden of morbidities among such children, which go unaddressed in the absence of MAM treatment programs (12).

Both in-hospital and post-discharge, mortality has been shown to increase dramatically with wasting status, with severely wasted children at a nearly six-fold increased risk of death and moderately wasted children a nearly three-fold increased risk of death compared to not wasted children (13). A high volume of costlier hospitalizations also puts enormous pressure on fragile health systems with limited capacity for delivering quality inpatient care due to a lack of diagnostics, treatments, and human and financial resources.

Niger routinely records some of the worst malnutrition indicators in the world (14, 15), a trend projected to continue for the foreseeable future (16). From 2014 to 2019, between 300 and 400,000 children were treated each year for SAM, of whom 11–15% required hospital care, with up to two-thirds of this caseload localized in the Maradi and Zinder regions (17). The extreme seasonality of admissions is likely driven by malaria incidence, but also points to a lack of access to diets of sufficient quality and quantity. Few households, for example, can afford a nutritious diet based on locally available foods (18), and only between 5% and 27% of children 6–23 months in Maradi and Zinder access a minimally acceptable diet that meet their nutritional needs (14, 15), showing the importance of preventive strategies in this context.

Optimizing MAlnutrition treatment (OptiMA) is a strategy for simplifying treatment protocols that is being tested in several operational pilots and two individually randomized control trials (RCTs) (19–21). OptiMA integrates the treatment for severe and moderate acute malnutrition in one program using only MUAC or oedema for admission and one treatment product – RUTF – at a decreasing dose as a child’s nutritional status improves. In 2019, a pilot study of OptiMA was conducted in two health zones of Mirriah District, Zinder Region, Niger. From 2015 to 2019 a parallel program in the same district aimed to reduce the incidence of acute malnutrition in children 6–23 months via supplementation with small quantity lipid-based nutritional supplement (SQ-LNS, brand name Enov’Nutributter™, 20 g/day) for up to 18 months. SQ-LNS supplementation in this age group has shown significant, simultaneous impacts on childhood malnutrition, including reductions in wasting, stunting, anemia, and mortality as well as improved developmental outcomes (22–25). This distribution remained in effect until December 2019 in one of the two health zones selected for the OptiMA pilot. The other OptiMA health zone did not receive the SQ-LNS distribution.

The aim of this study was to evaluate the program outcomes of a simplified protocol for treatment of children with acute malnutrition in a high burden context in Niger. A second objective was to identify the factors associated with hospitalization. Finally, this study was also conducted to inform the preparation of a recently completed OptiMA individual randomized control trial (RCT) in the same district of Niger (21).

## 2. Materials and methods

### 2.1. Study design, location and period

This prospective observational cohort study was conducted in two health zones of Mirriah Health District, Zinder Region from July to December 2019. The Alliance for Medical Action (ALIMA) and its partner organization in Niger, Bien-etre de la Femme et de l’Enfant (BEFEN) have supported the Ministry of Health (MoH) in Mirriah district since 2009 to provide medical services to women and children under 5 years of age. This has included continuous support for an 80-bed unit at the Mirriah District Hospital for treatment of pediatric emergencies and malnourished children with medical complications. Mirriah had a total of 19 health zones in 2019 each with a primary health center (PHC) providing a standard package of care including treatment of SAM and MAM with an established, UNICEF-supported RUTF supply chain. In 2019, ALIMA/BEFEN supported 10 PHCs, 2 of which were selected for this study.

Between 2015–19, ALIMA/BEFEN conducted a malnutrition prevention program that included support to the Expanded Program on Immunizations (EPI) and a monthly distribution of SQ-LNS among children under 2 years old in 3 health zones, including one of the two health zones selected for this study. Thus, a proportion of children identified with acute malnutrition in this study had received preventive nutritional supplementation prior to inclusion.

The population in Mirriah District was estimated at 700,000 inhabitants in 2019 with the population of both health areas in this pilot at 40,000 each. A SMART survey conducted in September 2019 showed rates of Global Acute Malnutrition (GAM) by MUAC of 8.2% and SAM of 2.6% (26).

### 2.2. Eligibility criteria, treatment and follow-up, and discharge criteria

**Figure 1:**
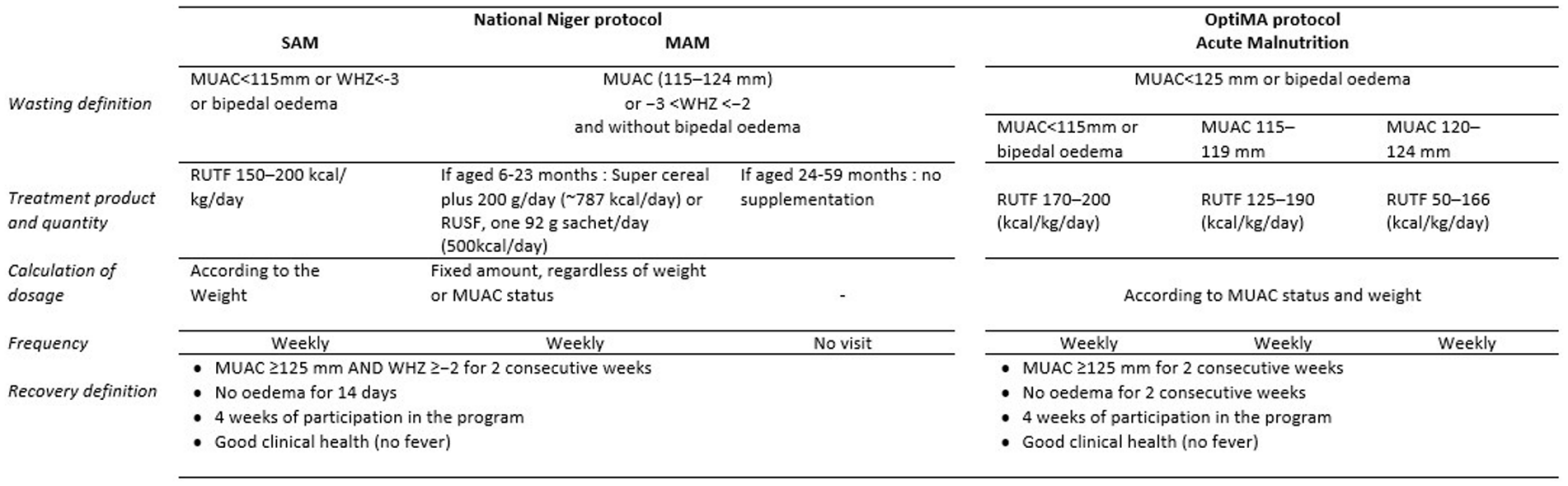
Comparison of standard CMAM protocol currently in use in Niger versus the OptiMA protocol, Mirriah, Niger 2019.

Figure 1 summarizes the differences between the current national treatment protocol in Niger and OptiMA. Children were considered eligible for enrollment if they were aged 6–59 months and presented spontaneously to either outpatient clinic in the two health zones with a MUAC <125 mm or oedema +/++ without medical complications and passed an appetite test on the day of inclusion.

Children were followed at weekly consultations at primary health care facilities, where they received a clinical exam that included anthropometric measurements, and given a weekly RUTF ration relative to the severity of their malnutrition. In contrast to the weight-based RUTF ration in the national program, which is fixed at 150–200 kcal/kg per d (628–837 kJ/kg per day) for the course of treatment, the OptiMA RUTF ration was calibrated to the child’s degree of wasting based on the combination of MUAC status and weight. Thus, more nutritional support was given to the most severely malnourished and gradually reduced as the child’s MUAC and weight increased. Children with MUAC <115 mm or oedema received 170–200 kcal/ kg per day of RUTF, while children with MUAC 115–119 mm, either at admission or during the course of treatment, received 125–190 kcal/kg per day, and children with MUAC≥120 mm received 50–166 kcal/kg per day (with a minimum of one sachet per day) until discharge from the program.

All children underwent malaria rapid testing upon inclusion and at any point during their follow-up if clinical signs of malaria were detected. All children with a positive malaria rapid diagnostic test were prescribed an artemisinin-combination treatment. Amoxicillin 90 mg/kg per day for 7 days was prescribed for all children with MUAC <120 mm or oedema at admission. Albendazole was given to children 9 months of age or older at the fourth follow-up visit if they had no deworming in the previous 4 months. Community health workers visited the homes of children who missed multiple consultations.

The criteria for ending the intervention was MUAC ≥125 mm and no oedema for two consecutive weeks (with a minimum stay in the program for 4 weeks). As a safety precaution, children whose anthropometry was WHZ < -3 and/or MUAC <115 mm after 10 weeks of treatment with the OptiMA protocol (rather than the normal 12 weeks) were transferred to the national protocol. Children with a MUAC ≥125 mm and WHZ < −3 at inclusion were managed instead according to the current national protocol; similarly, children with a MUAC ≥125 mm and WHZ between −2 and − 3 were managed by the national protocol for children with MAM.

### 2.3. Study outcomes

Primary outcomes were standard acute malnutrition program indicators (27), i.e., proportions of children 1. recovered (defined as a 4-week minimum duration of treatment, an axillary temperature < 37.5°C, and an absence of bipedal oedema and a MUAC ≥125 mm for two consecutive weeks), 2. defaulted (defined as a child absent for three consecutive consultations), 3. non-responders (defined as failure to reach recovery criteria after 10 weeks for children included with SAM and 12 weeks for those included with MAM), and 4. hospitalized.

Additional outcomes were 1. median number of sachets distributed to children who recovered, stratified by MUAC category upon inclusion; 2. adherence of health workers to the OptiMA RUTF dosage table; 3. MUAC gain (mm/d), weight gain (g/kg/d), and 4. length of hospitalization.

### 2.4. Data collection procedures and monitoring

Sociodemographic, clinical, and anthropometric data were collected by MoH staff supervised by a project manager using the national program individual outpatient record. A specific form was used for hospitalization data. The staff involved in data collection were trained on the study protocol before participant’s enrollment. The child’s weight, MUAC, temperature, clinical symptoms and amount of RUTF ration were recorded at each weekly visit. Children’s length was measured at admission and once a month thereafter. Weight was measured to the nearest 100 g with a Salter scale, and length was measured to the nearest 0·5 cm on a height board with the child in a supine position (or standing if taller than 85 cm). MUAC was measured to the nearest mm with a MUAC bracelet demarcated in 1 mm increments. At each visit, supervisors ensured that scales were correctly calibrated and MUAC bracelets and height boards were in good condition. All collected data was then anonymized before being entered into a database created, managed, and monitored by a binational research team based in France and Cote d’Ivoire. An external monitoring visit was carried out by the team at both study sites. Whether a child received SQ-LNS before inclusion was reported by the caretaker without specifying the duration of supplementation.

### 2.5. Data analysis

Children who had no visits beyond initial inclusion were excluded from analysis. Baseline characteristics of children included in the analysis were described at baseline by MUAC category at admission. Continuous variables were described in terms of mean (standard deviation, sd). Categorical variables were described in terms of frequency.

Study outcomes (program indicators, as described above) were described overall and stratified by MUAC category at admission with their 95%CI, and considered at least as effective as the international SPHERE standards if the lower limit of the 95%CI was greater than or equal to the reference value (28).

The association between hospitalization and the other variables was analyzed using logistic regression based on the Hosmer and Lemeshow test. All variables in univariate analysis with a value of *p* ≤0.25 were included in the multivariate analysis and variables with a value of *p* <0.05 were considered as statistically significant in the final model.

The adherence to the OptiMA RUTF dosage table was evaluated by the difference between weekly RUTF ration provided to the child and the theoretical ration as calculated by the OptiMA dosage table. MUAC gain was calculated in mm/day and weight gain was calculated in g/kg/day.

All analyses were conducted using SAS version 9.3 (SAS Institute Inc.).

### 2.6. Ethics

The study was approved by Niger’s National Ethics Committee for Health Research (Number 018/2019/CNERS July 11, 2019). Caregivers gave written consent prior to enrollment for all children included in the study. All data were anonymized when entered into the database, and unique identification numbers were coded.

## 3. Results

**Figure 2:**
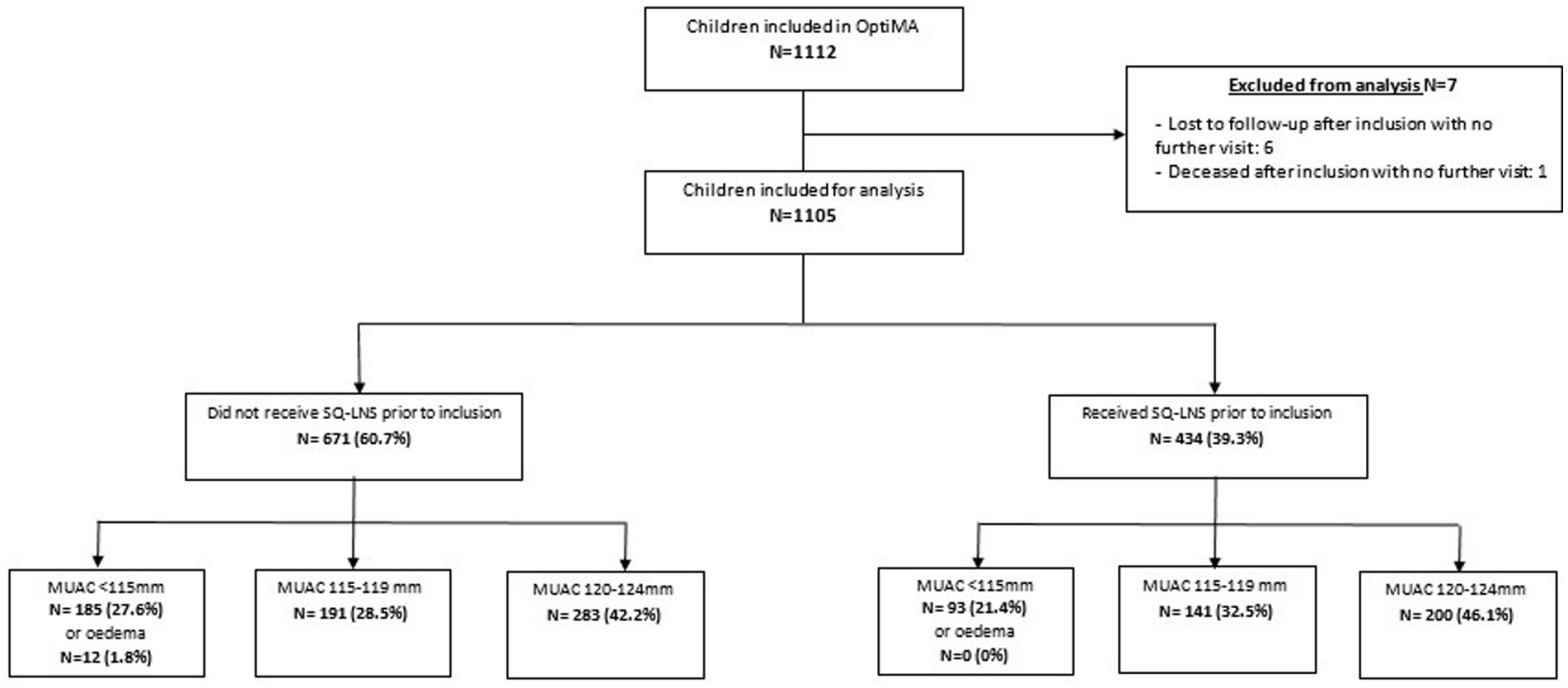
Flux diagram of children included for treatment under the OptiMA protocol, Mirriah, Niger 2019.

Between 22 July and 15 November 2019, a total of 1,112 children were included in this study under the OptiMA protocol, and 1,105 were included in the analysis (Figure 2). The 7 children omitted from analysis either only had the inclusion visit (*n* = 6) or were deceased (*n* = 1) without having any visits after admission.

Table 1 presents the characteristics at baseline. Among the 1,105 children included in the analysis, 12 (1.1%) were admitted with edema, 278 children (25.2%) with MUAC <115 mm, 332 (30.0%) with MUAC 115–119 mm, and 483 (43.7%) with MUAC 120–124 mm. Most of the children were female (55.5%) and under 24 months of age (85.4%). A total of 434 children (39.3%) received SQ-LNS prior to their inclusion for treatment, and all 12 children included with edema came from the group that had not received SQ-LNS. Stunting, or height-for-age z score (HAZ) of <−2, was observed in 79.1% of children, including more than half (51.4%) with severe stunting, and 63.1% of children presented with concomitant wasting (WHZ < −2) and stunting (HAZ < −2). Most of the children’s caretakers (62.4%) reported having received training for how to use a MUAC bracelet.

**Table 1:** Description of children included in optimizing treatment for acute malnutrition (OptiMA) protocol, Mirriah district, Niger, 2019 (Numbers and percentages; mean values and standard deviations).

Characteristics	Oedema +/++ *N* = 12 (1.1%)	MUAC < 115 mm *N* = 278 (25.2%)	MUAC 115–119 mm *N* = 332 (30.0%)	MUAC 120–124 mm *N* = 483 (43.7%)	Global *N* = 1,105
Sex, *n* (%)					
Girls	6 (50.0)	147 (52.9)	184 (55.4)	271 (56.1)	608 (55.0)
Boys	6 (50.0)	131 (47.1)	148 (44.6)	212 (43.9)	497 (45.0)
Age, mean (ET), months	21.7 (4.8)	13.9 (6.9)	15.1 (6.8)	16.0 (6.5)	15.2 (6.8)
Class age, *n* (%)					
6–12 months	1 (8.3)	158 (56.8)	156 (47.0)	185 (38.3)	500 (45.2)
13–23 months	5 (41.7)	85 (30.6)	129 (38.9)	225 (46.6)	444 (40.2)
≥ 24 months	6 (50.0)	35 (12.6)	47 (14.1)	73 (15.1)	161 (14.6)
Weight, mean (ET), kg	7.8 (0.5)	5.8 (1.0)	6.4 (0.8)	6.9 (0.9)	6.5 (1.0)
Height, mean (ET), cm	75.2 (2.2)	67.1 (5.6)	69.0 (5.2)	71.0 (5.4)	69.4 (5.6)
MUAC, mean (ET), mm	120.8 (4.8)	109.5 (4.9)	116.9 (1.4)	121.4 (1.4)	117.1 (5.5)
Weight/height, *n* (%), *z* score					
< −3	3 (25.0)	154 (55.4)	103 (31.0)	77 (16.0)	337 (30.5)
≥ − 3 et < −2	5 (41.7)	105 (37.8)	161 (48.5)	272 (56.3)	543 (49.1)
≥ − 2	4 (33.3)	19 (6.8)	68 (20.5)	134 (27.7)	225 (20.4)
Weight/age, *n* (%), *z* score					
< −3	7 (58.4)	260 (93.5)	260 (78.3)	264 (54.7)	791 (71.6)
≥ − 3 et < −2	4 (33.3)	18 (6.5)	65 (19.6)	185 (38.3)	272 (24.6)
≥ − 2	1 (8.3)	–	7 (2.1)	34 (7.1)	42 (3.8)
Height/age, *n* (%), *z* score					
< −3	8 (66.7)	175 (62.9)	179 (53.9)	206 (42.6)	568 (51.4)
≥ − 3 et < −2	3 (25.0)	67 (24.1)	101 (30.4)	135 (28.0)	306 (27.7)
≥ − 2	1 (8.3)	36 (13.0)	52 (15.7)	142 (29.4)	231 (20.9)
Children wasted and stunted, *n* (%)					
No	5 (41.7)	53 (19.1)	112 (33.7)	238 (49.3)	408 (36.9)
Yes	7 (58.3)	225 (80.9)	220 (66.3)	245 (50.7)	697 (63.1)
Fever (T > 38°C), *n* (%)					
No	12 (100)	257 (92.5)	307 (92.5)	450 (93.2)	1,026 (92.8)
Yes	–	21 (7.5)	25 (7.5)	33 (6.8)	79 (7.2)
Malaria rapid diagnostic test (RDT), *n* (%)					
Negative	7 (58.3)	241 (86.7)	278 (83.7)	410 (84.9)	36 (84.7)
Positive	5 (41.7)	37 (13.3)	54 (16.3)	73 (15.1)	169 (15.3)
Appetite test, *n* (%)					
Good	12 (100)	250 (89.9)	320 (96.4)	464 (96.1)	1,046 (94.7)
Average	–	28 (10.1)	12 (3.6)	19 (3.9)	59 (5.3)
Received SQ-LNS prior to inclusion, *n* (%)					
No	12 (100)	185 (66.6)	191 (57.5)	283 (58.6)	671 (60.7)
Yes	–	93 (33.4)	141 (42.5)	200 (41.4)	434 (39.3)
Breastfeeding, *n* (%)					
No	10 (83.3)	70 (25.2)	114 (34.3)	167 (34.6)	361 (32.7)
Yes	2 (16.7)	208 (74.8)	218 (65.7)	316 (65.4)	744 (67.3)
Received SMC, *n* (%)					
No	3 (25.0)	37 (13.3)	22 (6.6)	27 (5.6)	89 (8.1)
Yes	9 (75.0)	241 (86.7)	310 (93.4)	456 (94.4)	1,016 (91.9)
Received systematic amoxicillin treatment, *n* (%)					
No	–	–	–	475 (98.3)	475 (43.0)
Yes	12 (100)	278 (100)	332 (100)	8 (1.7)	630 (57.0)
Caretaker received MUAC training, *n* (%)					
No	6 (50.0)	109 (39.2)	122 (36.8)	179 (37.1)	416 (37.6)
Yes	6 (50.0)	169 (60.8)	210 (63.2)	304 (62.9)	689 (62.4)
MUAC bracelet in home, *n* (%)					
No	7 (58.3)	130 (46.8)	162 (48.8)	220 (45.5)	519 (47.0)
Yes	5 (41.7)	148 (53.2)	170 (51.2)	263 (54.5)	586 (53.0)

Description of program outcomes for children included in the analysis under OptiMA are presented in Table 2 and stratified by the WHO definition of SAM (i.e., MUAC<115 mm or WHZ < −3 or oedema) and MAM (MUAC [115–124] or WHZ [<−2 and ≥ −3] and no oedema) in Table 3. The overall 82.3% recovery rate of children treated with the OptiMA protocol exceeds SPHERE standards, however there were significant differences among MUAC categories at inclusion. The overall mortality, non-response and default rates were 0.6%, 12.6%, and 4.2%, respectively.

**Table 2:** Description of program outcomes stratified by MUAC or oedema at inclusion, OptiMA-Mirriah, Niger, 2019.

Characteristics	Oedema +/++ *N* = 12	MUAC < 115 mm *N* = 278	MUAC 115–119 mm *N* = 332	MUAC 120–124 mm *N* = 483	Global *N* = 1,105	*p*-value
Length of stay, weeks						<0.0001
Mean (sd)	5.5 (3.2)	6.7 (2.5)	5.7 (2.4)	4.9 (1.8)	5.6 (2.3)	
CI_95%_	[3.5–7.5]	[6.4–7.1]	[5.5–6.0]	[4.7–5.1]	[5.5–5.8]	
At least one hospitalization, *n* (%)						<0.001
No	9 (75.0)	206 (74.1)	285 (85.8)	438 (90.7)	938 (84.9)	
CI_95%_	[50.5–99.5]	[69.0–79.3]	[82.1–89.6]	[88.1–93.3]	[82.8–87.0]	
Yes	3 (25.0)	72 (25.9)	47 (14.2)	45 (9.3)	167 (15.1)	
CI_95%_	[0.5–49.5]	[20.7–31.1]	[10.4–17.9]	[6.7–11.9]	[13.0–17.2]	
Length of hospitalization, days						0.87
Mean (sd)	5.0 (1.7)	4.3 (1.9)	4.2 (1.1)	4.2 (1.5)	4.3 (1.6)	
CI_95%_	[0.7–9.3]	[3.9–4.8]	[3.9–4.6]	[3.8–4.7]	[4.0–4.5]	
Program Outcomes						<0.0001
Recovered	9 (75.0)	158 (56.8)	290 (87.3)	452 (93.6)	909 (82.3)	
CI_95%_	[50.0–99.5]	[51.0–62.7]	[83.8–90.9]	[91.4–95.8]	[80.0–84.5]	
Non-response	1 (8.3)	98 (35.3)	22 (6.6)	18 (3.7)	139 (12.6)	
CI_95%_	[0.0–24.0]	[29.6–40.9]	[3.9–9.3]	[2.0–5.4]	[10.6–14.5]	
Deceased	–	2 (0.7)	5 (1.5)	–	7 (0.6)	
CI_95%_		[0.0–1.7]	[0.2–2.8]		[0.2–1.1]	
Transfer	–	1 (0.4)	–	1 (0.2)	2 (0.2)	
CI_95%_		[0.0–1.1]		[0.0–0.6]	[0.0–0.4]	
Default	2 (16.7)	18 (6.5)	15 (4.5)	12 (2.5)	47 (4.2)	
CI_95%_	[0.0–37.7]	[3.6–9.4]	[2.3–6.7]	[1.1–3.9]	[3.1–5.4]	
Follow-up in progress	–	1 (0.3)	–	–	1 (0.1)	
CI_95%_		[0.0–1.1]			[0.0–0.3]	
Weight gain, mean (sd), g/kg/d						0.15
Global	3.7 (2.1)	4.3 (2.2)	4.3 (2.4)	4.0 (2.0)	4.2 (2.2)	
CI_95%_	[2.3–5.0]	[4.0–4.5]	[4.1–4.6]	[3.8–4.2]	[4.0–4.3]	
Among recovered	4.3 (2.0)	5.0 (1.8)	4.4 (2.1)	4.1 (2.0)	4.4 (2.0)	
CI_95%_	[2.7–5.8]	[4.7–5.3]	[4.2–4.7]	[3.9–4.3]	[4.2–4.5]	
Among non-responders	2.6	3.3 (1.9)	2.8 (2.2)	2.1 (1.5)	3.1 (2.0)	
IC_95%_		[2.9–3.7]	[1.8–3.8]	[1.4–0.8]	[2.8–3.4]	
MUAC gain, Mean (sd), mm/d						<0.0001
Global	0.14 (0.11)	0.25 (0.16)	0.23 (0.15)	0.19 (0.12)	0.22 (0.14)	
CI_95%_	[0.07–0.22]	[0.23–0.27]	[0.21–0.24]	[0.18–0.20]	[0.21–0.22]	
Among recovered	0.17 (0.12)	0.33 (0.13)	0.25 (0.11)	0.20 (0.10)	0.24 (0.12)	
CI_95%_	[0.08–0.26]	[0.31–035]	[0.24–0.27]	[0.19–0.21]	[0.23–0.25]	
Among non-responders	0.07	0.14 (0.10)	0.03 (0.05)	−0.02 (0.06)	0.10 (0.11)	
CI_95%_		[0.12–0.16]	[0.01–0.05]	[−0.05–0.01]	[0.08–0.12]	
RUTF received, Mean (sd), sachets						<0.0001
Global	90.5 (29.8)	100.2 (32.6)	67.6 (25.0)	49.2 (18.3)	68.0 (32.1)	
CI_95%_	[71.6–109.4]	[96.3–104.0]	[64.9–70.3]	[47.6–50.8]	[66.1–69.9]	
Among recovered	87.4 (29.6)	85.8 (23.6)	65.9 (21.0)	47.5 (13.7)	60.4 (23.4)	
CI_95%_	[64.7–110.2]	[82.1–89.5]	[63.5–68.4]	[46.2–48.8]	[58.9–62.0]	
Among non-responders	137	131.2 (19.8)	114.3 (22.7)	105.4 (24.9)	125.3 (23.0)	
CI_95%_	–	[127.3–135.2]	[104.2–124.4]	[93.0–117.8]	[121.4–129.1]	

**Table 3:** Description of program outcomes stratified by WHO definition for SAM and MAM at admission, OptiMA-Mirriah, Niger, 2019.

Characteristics	SAM by WHO definition (WHZ < ˗3Z and/or MUAC < 115 mm and/or presence of edema)		Global *N* = 1,105	
	No *N* = 635	Yes *N* = 470		*p*-value
Length of stay, weeks				<0.001
Mean (sd)	5.2 (2.0)	6.2 (2.5)	5.6 (2.3)	
CI_95%_	[5.1–5.4]	[5.9–6.4]	[5.5–5.8]	
At least one hospitalization, *n* (%)				<0.0001
No	564 (88.8)	374 (79.6)	938 (84.9)	
Yes	71 (11.2)	96 (20.4)	167 (15.1)	
Length of hospitalization, days				0.44
Mean (sd)	4.2 (1.4)	4.4 (1.8)	4.3 (1.6)	
CI_95%_	[3.8–4.5]	[4.0–4.7]	[4.0–4.5]	
Outcome				<0.0001
Recovered	578 (91.0)	331 (70.4)	909 (82.3)	
Non-response	32 (5.0)	107 (22.8)	139 (2.6)	
Deceased	4 (0.6)	3 (0.6)	7 (0.6)	
Transfer	1 (0.2)	1 (0.2)	2 (0.2)	
Default	20 (3.1)	27 (5.7)	47 (4.2)	
Follow-up in progress	–	1 (0.2)	1 (0.1)	
Weight gain, mean (sd), g/kg/d				<0.0001
Global	3.9 (1.9)	4.5 (2.5)	4.2 (2.2)	
CI_95%_	[3.8–4.1]	[4.3–4.7]	[4.0–4.3]	
Among recovered	4.0 (1.8)	5.0 (2.3)	4.4 (2.0)	
CI_95%_	[3.9–4.2]	[4.7–5.2]	[4.2–4.5]	
Among non-responders	2.4 (1.7)	3.3 (2.0)	3.1 (2.0)	
IC_95%_	[1.8–3.1]	[2.9–3.7]	[2.8–3.4]	
MUAC gain, Mean (sd), mm/d				<0.0001
Global	0.20 (0.12)	0.24 (016)	0.22 (0.14)	
CI_95%_	[0.19–0.21]	[0.22–0.25]	[0.21–0.22]	
Among recovered	0.21 (0.11)	0.28 (0.13)	0.24 (0.12)	
CI_95%_	[0.21–0.22]	[0.27–0.30]	[0.23–0.25]	
Among non-responders	0.01 (0.06)	0.13 (0.10)	0.10 (0.11)	
CI_95%_	[−0.02–0.03]	[0.11–0.15]	[0.08–0.12]	
RUTF received, Mean (sd), sachets				<0.0001
Global	56.3 (23.3)	83.8 (35.5)	68.0 (32.1)	
CI_95%_	[54.5–58.2]	[80.5–87.0]	[66.1–69.9]	
Among recovered	54.1 (19.0)	71.5 (26.2)	60.4 (23.4)	
CI_95%_	[52.5–55.6]	[68.7–74.4]	[58.9–62.0]	
Among non-responders	111.5 (22.9)	129.4 (21.5)	125.3 (23.0)	
CI_95%_	[103.3–119.7]	[125.2–133.5]	[121.4–129.1]	

Recovery was lowest (57.6%) and non-response highest (34.1%) for children with MUAC <115 mm and/or oedema at admission, while mortality remained low (0.7%). Overall length of stay in the program was 5.6 weeks, and 6.7 weeks for children admitted with MUAC <115 mm. Among all recovered children and those admitted with MUAC <115 mm and/or oedema, MUAC gain was 0.24 and 0.33 mm per day, weight gain was 4.4 and 5.0 g/kg per day, and the average amount of RUTF received was 60.4 and 85.8 sachets, respectively.

A total of 167 children (15.1%) required at least one hospital stay during the course of treatment, including 75/290 (25.9%) children admitted with MUAC <115 mm and/or oedema and 92/815 (11.3%) of children admitted with MUAC 115 to 124 mm. Children meeting the current WHO definition of SAM or MAM were hospitalized at rates of 20.4 and 11.2%, respectively. Three of the 7 total deaths occurred in hospital, for an inpatient mortality rate of 1.8%. One child died a week after being discharged from hospital.

Table 4 shows factors associated with hospitalization. In multivariate analysis, factors significantly associated with hospitalization were a child having MUAC<115 mm and/or edema (adjusted odds ratio 3.4; 95% CI 2.01–4.60), being younger than 2 years of age (adjusted OR 3.69; 95% CI 1.85–7.33 for children 6–12 m, and adjusted OR 2.28; 95% CI 1.12–4.65 for children 13–23 m), or presenting with a fever (adjusted OR 1.88; 95% CI 1.21–2.93). Adjusted for gender, age, and MUAC status, having received the SQ-LNS before inclusion was found to be protective, reducing the risk of hospitalization by 43% (adjusted OR 0.57; CI at 95% 0.39–0.84).

**Table 4:** Logistic regression analysis of factors associated with hospitalization during treatment, OptiMA-Mirriah, Niger, 2019.

Characteristics at inclusion	Univariate analysis					Multivariate analysis		
	No hospital (*n* = 938)	Hospital (*n* = 167)	OR	IC_95%_	*P*	OR	IC_95%_	*p*
MUAC at inclusion, *n* (%)					<0.001			<0.001
MUAC < 115 and/or edema +/++	215 (22.9)	75 (44.9)	3.40	[2.27–5.09]		3.04	[2.01–4.60]	
MUAC 115–119	285 (30.4)	47 (28.1)	1.61	[1.04–2.48]		1.53	[0.98–2.38]	
MUAC 120–124	438 (46.7)	45 (27.0)	1	–		1	–	
Sex, *n* (%)					0.60			
Girl	513 (54.7)	95 (56.9)	1	–				
Boy	425 (45.3)	72 (43.1)	0.92	[0.66–1.28]				
Age, mean (ET), months	15.7 (6.8)	12.9 (6.0)	0.93	[0.91–0.96]	<0.001			
Age, *n* (%)					<0.001			0.0002
6–12 months	396 (42.2)	104 (62.3)	4.00	[2.02–7.79]		3.69	[1.85–7.33]	
13–23 months	391 (41.7)	53 (31.7)	2.05	[1.02–4.13]		2.28	[1.12–4.65]	
≥ 24 months	151 (16.1)	10 (6.0)	1	–		1	–	
Weight-for-height, *n* (%), *Z* score					0.02			
< −3	271 (28.9)	66 (39.5)	1.42	[0.90–2.24]				
≥ − 3 and < −2	475 (50.6)	68 (40.7)	0.83	[0.53–1.30]				
≥ − 2	192 (20.5)	33 (19.8)	1	–				
Weight-for-age, *n* (%), *Z* score					0.46			
< −3	666 (71.0)	125 (74.8)	1.78	[0.63–5.08]				
≥ − 3 and < −2	234 (25.0)	38 (22.8)	1.54	[0.52–4.57]				
≥ − 2	38 (4.0)	4 (2.4)	1	–				
Height-for-age, *n* (%), *Z* score					0.70			
< −3	481 (51.3)	87 (52.1)	1.17	[0.75–1.82]				
≥ − 3 et < −2	257 (27.4)	49 (29.3)	1.23	[0.76–2.00]				
≥ − 2	200 (1.3)	31 (18.6)	1	–				
Fever during stay (>38°C), *n* (%)					0.007			0.005
No	821 (87.5)	133 (79.6)	1	–		1	–	
Yes	117 (12.5)	34 (20.4)	1.79	[1.17–2.74]		1.88	[1.21–2.93]	
Breastfeeding, *n* (%)					0.001			
No	325 (34.7)	36 (21.6)	1	–				
Yes	613 (63.3)	131 (78.4)	1.93	[1.30–2.86]				
SMC received, *n* (%)					0.04			
No	69 (7.4)	20 (12.0)	1	–				
Yes	869 (92.6)	147 (88.0)	0.58	[0.34–0.99]				
Received systematic amoxocillin treatment at inclusion, *n* (%)					<0.001			
No	430 (45.8)	45 (27.0)	1	–				
Yes	508 (54.2)	122 (73.0)	2.29	[1.59–3.31]				
SQ-LNS received prior to inclusion, *n* (%)					0.0002			0.004
No	548 (58.4)	123 (73.6)	1	–		1	–	
Yes	390 (41.6)	44 (26.4)	0.50	[0.35–0.73]		0.57	[0.39–0.84]	
MUAC training received, *n* (%)					0.11			
No	344 (36.7)	72 (43.1)	1	–				
Yes	594 (63.3)	95 (56.9)	0.76	[0.55–1.07]				
MUAC bracelet at home, *n* (%)					0.27			
No	434 (46.3)	85 (50.9)	1	–				
Yes	504 (53.7)	82 (49.1)	0.83	[0.60–1.16]				

**Figure 3:**
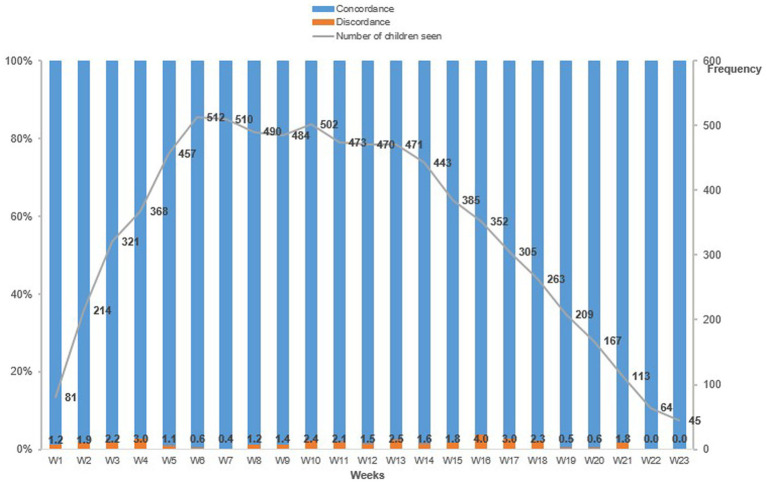
Weekly concordance and discordance to the theoretical number of RUTF sachets to be distributed by health personnel to children under the OptiMA protocol, Mirriah, Niger 2019.

We observed a weekly proportion of errors in RUTF distribution by health workers ranging from 0% to 4% throughout the 27 weeks of the study, including 2.1% among the 1,105 children at the inclusion visit, 1.7% out of 6,629 total visits for the follow-up consultations (Figure 3).

## 4. Discussion

This prospective observational cohort study evaluated a therapeutic nutrition protocol for children affected by acute malnutrition, defined as MUAC <125 mm or oedema, and treated with one product (RUTF) at a gradually reduced dose based on a child’s weight and MUAC status. Overall recovery rates were acceptable, but low rates among children included with MUAC <115 mm or edema are concerning. An average of 68.8 sachets of RUTF were distributed per child per course of treatment, with very few (1.7%) ration errors. An unexpected finding was that children who received SQ-LNS prior to inclusion were 43% less likely to be hospitalized during treatment for acute malnutrition with RUTF.

Recovery and mortality rates– 82·3% and 0·7%, respectively – exceed SPHERE standards and are in line with those reported by similar MUAC-based programs integrating SAM and MAM management with reduced RUTF dosage (5, 19, 28). Recovery among children admitted at MUAC <115 mm or oedema is concerning (56·8%), but was largely driven by high non-response (35·3%) and not mortality (0.7%). The recovery rate for children who met the current WHO definition of SAM was higher (70.4%) and non-response lower (22.8%). An analysis of children classified as non-responders will be reported elsewhere.

While lower recovery and higher non-response in the subgroup of children admitted with MUAC <115 mm requires further investigation, in a recent individually randomized control trial in the Democratic Republic of Congo, OptiMA demonstrated non-inferior among the subgroup of children included with the WHO definition of SAM (29). The ComPAS study, a cluster randomized trial in South Sudan and Kenya using similar admissions and discharge criteria but a different reduced RUTF dosage regimen, was non-inferior to the standard protocol in terms of recovery, but this study was not powered to assess recovery in children with MUAC <115 mm (6). Furthermore, the rates reported here are within the range reported in Niger and elsewhere in studies with and without RUTF dose reduction, suggesting that recovery for this category of children is challenging across contexts and may not be related to RUTF dosage (5–7, 30–33). A three-arm individual RCT comparing ComPAS, OptiMA, and Niger’s national standard was recently completed in the same district where this cohort occurred (21); like OptiMA-DRC, it was powered to compare outcomes of children with SAM and, for the first time, among children admitted with MUAC <115 mm.

Current SAM programs typically plan RUTF consumption between 120 and 150 sachets per child treated, while MAM programs plan between 60 and 90 sachets of ready-to-use supplementary food per child (34, 35). The average RUTF distribution of 68.8 sachets per child per course of treatment is slightly higher than the 60.8 sachets per child per course of treatment reported in the OptiMA pilot in Burkina Faso or the 64.0 sachets in the OptiMA-DRC trial, which perhaps reflects rural Niger’s heavier burden of malnutrition (19, 20). This could be because of the lower average MUAC at admission (117 mm) in this study compared to studies from Burkina Faso (119 mm) and DRC (120 mm).

In total, we found 1.7% of ration errors on 7,699 visits (inclusion and follow-up). This level of adherence is higher than that documented in Burkina Faso where a similar dosage table was used and a ration error was found for 24.1% of visits (19). This level of adherence was the result of one day of theoretical training for the health center staff followed by direct supervision by the doctor in charge of the two study sites.

The 15.1% rate of hospitalization is in line with Niger’s historical trend (17). More than 40% of hospitalized children were admitted with MAM, which is not surprising given a previous study showing substantial morbidities in such children (12). Inpatient mortality was 1.8%, well below the threshold of 10% used by the WHO (10). While interpretation of this low inpatient mortality rate should be done with caution, it may be related to the fact that OptiMA provides for early initiation of treatment in the MAM population, allowing clinicians to detect children who may need hospital care at an earlier stage of the wasting process. A recent study in Sierra Leone showed how treating children with MAM substantially lowered the risk of deteriorating to SAM and death when compared to nutrition counseling alone (36).

This operational study was unique in that nearly 40% of the children enrolled in OptiMA had been supplemented with SQ-LNS before being treated for acute malnutrition with RUTF. Routine supplementation with SQ-LNS to at-risk children from 6–23 months has a strong evidence base, including significant relative risk reductions in stunting, wasting, anemia, and mortality, as well as improved cognitive development (22–25). While the duration of SQ-LNS prior to inclusion under OptiMA was not recorded, this is the first investigation of how supplementation with SQ-LNS may articulate with simplified treatment protocols in a zone with an extremely high burden of acute malnutrition.

An important finding was that children who received SQ-LNS prior to being treated for malnutrition were 43% less likely to be hospitalized. This protection against hospitalization could result from the sustained access to high-quality complementary food helping these children to better weather an episode of acute malnutrition. Any combination of interventions that reduces hospitalizations needs serious consideration, especially in areas like southern Niger where inpatient care is costly to both individuals and the fragile public health system.

That none of the 12 children admitted under OptiMA with edema came from the SQ-LNS group is interesting. It is impossible to say if SQ-LNS had any preventive effect because the numbers are too small, and the health zone without SQ-LNS distributions is a known foyer for edema. There is, however, biological plausibility. Even though the etiology of kwashiorkor is unclear, evidence points to the lack of a balanced diet including animal source proteins is a likely contributing factor, and SQ-LNS can help fill in these nutrient gaps (37, 38). Testing this hypothesis in a context with a high burden of kwashiorkor would be a valuable future study.

There were two main limitations to this study. First, even though the percent positive for a rapid diagnostic test for malaria at inclusion is known, no data was collected on clinical diagnoses of malaria, diarrhea, pneumonia, or other co-morbidities such as HIV or tuberculosis during treatment. Adjusting the model with such variables could impact the association of SQ-LNS with reduced hospitalizations. Second, stratification of SAM and MAM according to the WHO definition does not reflect the burden of SAM and MAM according this definition but only among children included with MUAC <125 mm or edema.

One strength of this study is the robust data collection and monitoring conducted in a program setting that led to a high-quality individualized database. While this is not common in programs, we show here that it is possible. Another strength is the adherence to the protocol as reflected in the low rates of discordance in following the OptiMA RUTF distribution table and low rate of systematic antibiotic treatment for children admitted with a MUAC of 120–124 mm.

## 5. Conclusion

A simplified protocol in rural Niger resulted in acceptable recovery rates overall, but substantially lower recovery rates among children admitted <115 mm, in whom non-response was high. This requires further investigation. Supplementation with SQ-LNS before treatment was associated with fewer hospitalizations, showing how combining food-based prevention and simplified treatment protocols may yield benefits above and beyond what each intervention confers alone in areas with a persistently high burden of malnutrition.

## Data availability statement

The raw data supporting the conclusions of this article will be made available by the authors, without undue reservation.

## Ethics statement

The studies involving human participants were reviewed and approved by National Ethics Committee for Health Research in Niger (Number 018/2019/CNERS July 11, 2019). Written informed consent to participate in this study was provided by the participants’ legal guardian/next of kin.

## Author contributions

SS, KP, and RA designed the study methodology and wrote the protocol. RA, AA, OM, AS, and AM coordinated the study teams and Ministry of Health of Niger staff working in the trial. BS, RA, RM, AA, OM, and CY organized and supervised data collection. CY developed the database. BS, KP, MD, SS, and RM developed the statistical analysis strategy. BS performed the statistical analysis and all other authors interpreted the results. KP wrote the first draft of the manuscript with substantial inputs from SS, MD, and CC. KP, SS, and RB were primarily responsible for the final content of the manuscript. KP, BS, MD, CY, RA, AA, OM, AS, AM, CC, RM, RB, and SS critically reviewed the first draft and made substantial writing contributions to the development of the final manuscript and had full access to all the data in the study. BS, MD, CC, CY, and RM verified the underlying data of the study. KP, SS, and RB had final responsibility for the decision to submit the manuscript for publication. All authors contributed to the article and approved the submitted version.

## Funding

This study was primarily funded by the European Commission through the European Civil Protection and Humanitarian Aid Operations (ECHO, Brussels, Belgium). This Article covers humanitarian aid activities implemented with the financial assistance of the EU. The views expressed herein should not be taken, in any way, to reflect the official opinion of the EU, and the European Commission is not responsible for any use that might be made of the information it contains. UNICEF provided the ready-to-use therapeutic food (RUTF) for the study.

## Conflict of interest

KP serves on the Social Purposes Advisory Commission of Nutriset, a main producer of lipid-based nutrient supplement products.

The remaining authors declare that the research was conducted in the absence of any commercial or financial relationships that could be construed as a potential conflict of interest.

## Publisher’s note

All claims expressed in this article are solely those of the authors and do not necessarily represent those of their affiliated organizations, or those of the publisher, the editors and the reviewers. Any product that may be evaluated in this article, or claim that may be made by its manufacturer, is not guaranteed or endorsed by the publisher.

## Footnotes

^1^ kirrily.de.polnay@brussels.msf.org
